# Mitochondrial ROS prime the hyperglycemic shift from apoptosis to necroptosis

**DOI:** 10.1038/s41420-020-00370-3

**Published:** 2020-11-26

**Authors:** Matthew A. Deragon, William D. McCaig, Payal S. Patel, Robert J. Haluska, Alexa L. Hodges, Sergey A. Sosunov, Michael P. Murphy, Vadim S. Ten, Timothy J. LaRocca

**Affiliations:** 1 Department of Basic and Clinical Sciences, Albany College of Pharmacy and Health Sciences, Albany, NY 12208 USA; 2 Department of Pediatrics, Columbia University, New York, NY 10032 USA; 3 MRC Mitochondrial Biology Unit, Hills Road, Cambridge, CB2 0XY UK

**Keywords:** Necroptosis, Apoptosis

## Abstract

We have previously identified a shift from TNF-α-induced apoptosis to necroptosis that occurs under hyperglycemic conditions. This shift involves the downregulation or silencing of caspases and concurrent upregulation of necroptotic proteins leading to activation of the necrosome. In addition, under hyperglycemic conditions in vivo, this shift in cell death mechanisms exacerbates neonatal hypoxia-ischemia (HI) brain injury. Here, we identify two major factors that drive the hyperglycemic shift to necroptosis: (1) reactive oxygen species (ROS) and (2) receptor-interacting protein kinase 1 (RIP1). ROS, including mitochondrial superoxide, led to the oxidation of RIP1, as well as formation and activation of the necrosome. Concurrently, ROS mediate a decrease in the levels and activation of executioner caspases-3, -6, and -7. Importantly, hyperglycemia and mitochondrial ROS result in the oxidation of RIP1 and loss of executioner caspases prior to death receptor engagement by TNF-α. Moreover, RIP1 partially controlled levels of mitochondrial ROS in the context of hyperglycemia. As a result of its regulation of ROS, RIP1 also regulated necrosome activation and caspase loss. Mitochondrial ROS exacerbated neonatal HI-brain injury in hyperglycemic mice, as a result of the shift from apoptosis to necroptosis.

## Introduction

Necroptosis is the major pathway of programmed necrosis and has been documented to occur in diverse cell types, including erythrocytes, leukocytes, cardiac cells, and neurons^1–7^. This pathway is driven by the cytosolic necrosome complex, which consists of receptor-interacting protein kinase 1 (RIP1), RIP3, and mixed lineage kinase domain-like (MLKL) protein^1,8,9^. Downstream of this complex, MLKL oligomerizes and translocates to the cell membrane, forming pores^10–13^. In addition to MLKL, reactive oxygen species (ROS) are major effectors in necroptosis^8,14,15^. The formation of ROS during necroptosis is due to the interaction of necrosome components with several metabolic factors in the cell^16^. RIP3 may stimulate glycolysis and glutamate metabolism via activation of glycogen phosphorylase and glutamate ammonia ligase, respectively^17^. Moreover, RIP3 stimulates the citric acid cycle via activation of pyruvate dehydrogenase complex and glutamate dehydrogenase 1 (refs. ^17,18^). Stimulation at each of these metabolic steps ultimately leads to increased respiratory chain activity and ROS formation^16^. In addition, RIP1 has been shown to have a key role in direct stimulation of respiratory chain activity via activation of the transcriptional coactivator, peroxisome proliferator-activated receptor-gamma coactivator 1 alpha^19^. While ROS produce downstream cellular damage in necroptosis^14,15^, they have recently been shown to be critical for the oxidation and phosphorylation/activation of RIP1, inducing necroptosis and amplifying it through a feedback mechanism^20^.

In contrast to apoptosis, necroptosis results in a pro-inflammatory outcome^21–24^. Rather than removal of dying cells by macrophages, as is the case in apoptosis^25^, cells undergoing necroptosis lose membrane integrity and lyse, releasing all cellular contents^1,8,21^. Despite the vast differences in outcomes, there is some overlap in the initial steps of TNF-induced apoptosis and necroptosis^1,8,25^. Both pathways may be induced by TNF-α and involve the formation of membrane–proximal protein complexes^26–28^. Following this the signaling pathways diverge, with apoptosis driven by caspases and complex II and necroptosis driven by RIP1, RIP3, MLKL, and the necrosome^1,8,25^. That these pathways share induction steps and reach a point of divergence suggests that different cellular situations may favor one pathway over the other.

We previously reported that hyperglycemia/high-glucose conditions are a cellular situation that favors necroptosis over apoptosis^29^. That work demonstrated that high glucose potentiates a shift to RIP1-dependent necroptosis, despite specific stimulation of extrinsic apoptosis. In addition, we showed that this cell death shift is relevant to neonatal hypoxia-ischemia (HI)-brain injury in hyperglycemic mice^29^. At that time, we noted two factors on which this cell death shift depended: RIP1 and ROS^29^. In the current study, we aim to understand the underlying molecular mechanism of the hyperglycemic shift from apoptosis to necroptosis, with a primary focus on the role of RIP1 and ROS. We demonstrate that ROS are necessary and sufficient to produce the cell death shift from apoptosis to necroptosis. Furthermore, we show that ROS are partially dependent on RIP1 during the hyperglycemic shift to necroptosis. Importantly, ROS are also necessary for this cell death shift in vivo, as we show an exacerbation of neonatal HI-brain injury in hyperglycemic mice.

## Results

### The hyperglycemic shift from apoptosis to necroptosis depends on mitochondrial ROS

**Fig. 1:**
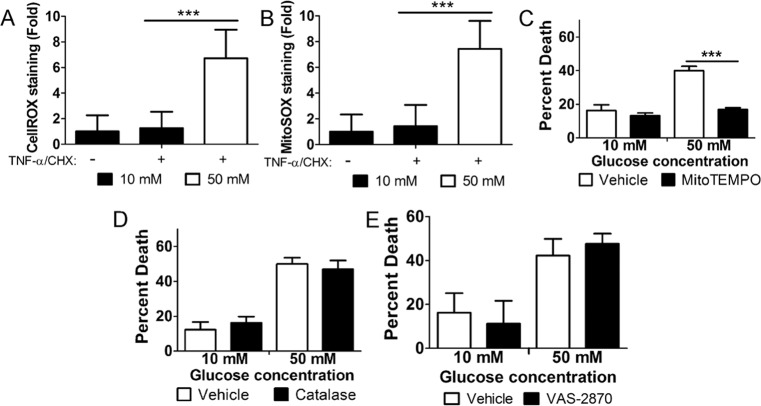
The hyperglycemic shift to necroptosis depends on mitochondrial ROS. U937 cells were grown in 10 or 50 mM glucose followed by the treatment with TNF-α/CHX for 4 h. Cells were then stained with **A** CellROX Green or **B** MitoSOX Red, and analyzed by flow cytometry. There is a robust increase in staining for both reagents in cells treated with TNF-α/CHX in 50 mM glucose. U937 cells were grown in 10 or 50 mM glucose followed by the treatment with TNF-α/CHX for 24 h in the presence of vehicle controls or **C** mitochondrial ROS scavenger, mitoTEMPO, **D** catalase, and **E** NADPH oxidase inhibitor, VAS-2870. WST-1 viability assays revealed that cell death in 50 mM glucose was prevented by mitoTEMPO and none of the other inhibitors. All results are from three independent experiments. Graphed values represent mean ± standard deviation. Two-way ANOVA, ***p < 0.001.

The hyperglycemic shift from apoptosis to necroptosis involves ROS^29^. We measured total cellular levels of oxidative stress, as well as mitochondrial ROS using CellROX Green and MitoSOX Red, respectively. For this, U937 cells were cultured in 10 or 50 mM glucose at 37 °C for 24 h, followed by the treatment with TNF-α/CHX for 6 h, staining, and flow cytometry. Both total cellular oxidative stress and mitochondrial ROS exhibited a robust increase in 50 mM glucose (Fig. 1A, B). Through the use of specific inhibitors, we probed the source of cellular ROS during this cell death shift. U937 monocytes were cultured in 10 or 50 mM glucose at 37 °C for 24 h followed by the treatment with TNF-α/CHX for 24 h in the presence or absence of various inhibitors. In line with our previous work, TNF-α/CHX-induced cell death increased in 50 mM glucose (Fig. 1C–F). Scavenging of mitochondrial ROS with mitoTEMPO^30,31^ prevented this increase in cell death (Fig. 1C). Scavenging of cellular hydrogen peroxide with catalase^32,33^, however, had no effect on cell death in 50 mM glucose (Fig. 1D). In addition, inhibition of NADPH oxidases, another source of ROS^34^, with VAS-2870 did not affect cell death in 50 mM glucose (Fig. 1E). These results suggest that mitochondrial ROS are the source that drives the hyperglycemic shift from apoptosis to necroptosis.

### ROS and RIP1 regulate one another during the hyperglycemic shift to necroptosis

**Fig. 2:**
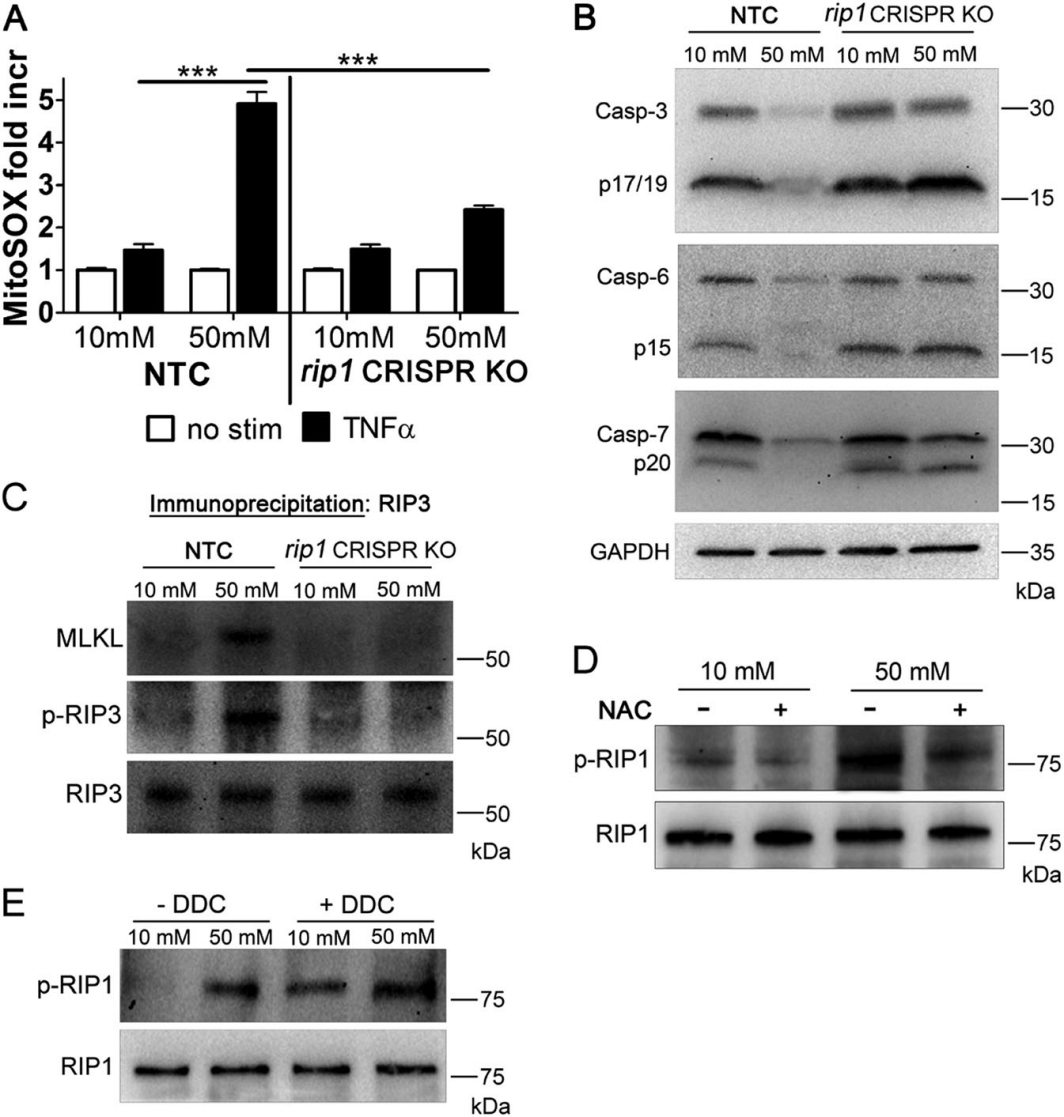
RIP1 and mitochondrial ROS act on one another during the hyperglycemic shift to necroptosis. **A** *rip1* CRISPR KO U937 cells or those transfected with nontargeting control guide RNA (NTC) were grown in 10 or 50 mM glucose, treated with TNF-α/CHX for 4 h followed by staining with MitoSOX Red and flow cytometry. The robust increase in MitoSOX Red staining in NTC cells in 50 mM glucose is blunted in *rip1* CRISPR KO cells. Results are from three independent experiments. Graphed values represent mean ± standard deviation. Two-way ANOVA, ***p < 0.001. **B** *rip1* CRISPR KO or NTC cells were grown in 10 or 50 mM glucose, treated with TNF-α/CHX for 2.5 h followed by lysate preparation and immunoblotting. Caspases-3, -6, and -7 decrease in NTC cells in 50 mM glucose, however, this decrease is not exhibited in *rip1* CRISPR KO cells. **C** *rip1* CRISPR KO or NTC cells were treated as in **B** followed by immunoprecipitation of RIP3 and immunoblotting. RIP3 is phosphorylated (p-RIP3) and MLKL co-precipitates in NTC cells in 50 mM glucose. Phosphorylation of RIP3 and co-precipitation of MLKL with RIP3 was not exhibited in *rip1* CRISPR KO cells. WT U937 cells were treated as in **B** in the presence or absence of **D** antioxidant, N-acetylcysteine (NAC) or **E** superoxide dismutase inhibitor, diethyldithiocarbamate (DDC). Total RIP1 levels were normalized. Phosphorylation of RIP1 (p-RIP1) is exhibited in 50 mM glucose and prevented by NAC. Treatment with DDC led to p-RIP1 in 10 or 50 mM glucose. All western blot images are representative of three independent experiments.

We previously established that RIP1 is a key factor in the hyperglycemic shift from apoptosis to necroptosis^29^. When the RIP1 gene is deleted in U937 monocytes they undergo caspase-dependent apoptosis in response to TNF-α/CHX under hyperglycemic conditions^29^. To probe the role of RIP1 further, we utilized a U937 cell line in which RIP1 was deleted using CRISPR-Cas9 (ref. ^29^), as well as U937 cells transfected with nonspecific guide RNA as a nontargeting control (NTC). These cells were grown in 10 or 50 mM glucose at 37 °C for 24 h, followed by the treatment with TNF-α/CHX for 6 h at 37 °C. Using mitoSOX Red and flow cytometry, we measured levels of mitochondrial ROS. MitoSOX Red staining increased by approximately fourfold in NTC cells in 50 mM glucose (Fig. 2A). However, *rip1* CRISPR KO cells only exhibited approximately twofold increase in mitoSOX staining in 50 mM glucose (Fig. 2A). This indicates that RIP1 partially regulates the increase in mitochondrial ROS during the hyperglycemic shift to necroptosis. In a separate set of experiments, the *rip1* CRISPR KO or NTC cells were grown in 10 or 50 mM glucose at 37 °C for 24 h, followed by the treatment with TNF-α/CHX for 2.5 h at 37 °C, lysate preparation, and western blotting. Total and activated levels of executioner caspases-3, -6, and -7 decrease in NTC cells in 50 mM glucose (Fig. 2B). This decrease did not occur in *rip1* CRISPR KO cells in 50 mM glucose (Fig. 2B). This shows that RIP1 is needed for the decrease in levels and activation of caspases during the hyperglycemic shift to necroptosis. In a similar set of experiments, RIP3 was immunoprecipitated and analyzed by western blot. RIP3 phosphorylation and co-precipitation of MLKL with RIP3 both increased in NTC cells in 50 mM glucose (Fig. 2C). However, this increase did not occur in *rip1* CRISPR KO cells in 50 mM glucose (Fig. 2C). This indicates that RIP1 is necessary for RIP3 phosphorylation and necrosome formation during the hyperglycemic shift to necroptosis. Interestingly, while RIP1 appears to regulate mitochondrial ROS during the hyperglycemic shift to necroptosis (Fig. 2A), ROS scavenging by N-acetylcysteine (NAC) resulted in a decrease in total and phosphorylated RIP1 in 50 mM glucose (Fig. 2D and Fig. S1A). Moreover, induction of ROS with the superoxide dismutase inhibitor, diethyldithiocarbamate (DDC), resulted in increased total levels and phosphorylation of RIP1, even in 10 mM glucose conditions (Fig. 2E and Fig. S1B). Collectively, these results indicate that while RIP1 regulates mitochondrial ROS during the hyperglycemic shift to necroptosis, it is reciprocally regulated by ROS.

### Reactive oxygen species activate necroptosis kinases while inhibiting caspases

**Fig. 3:**
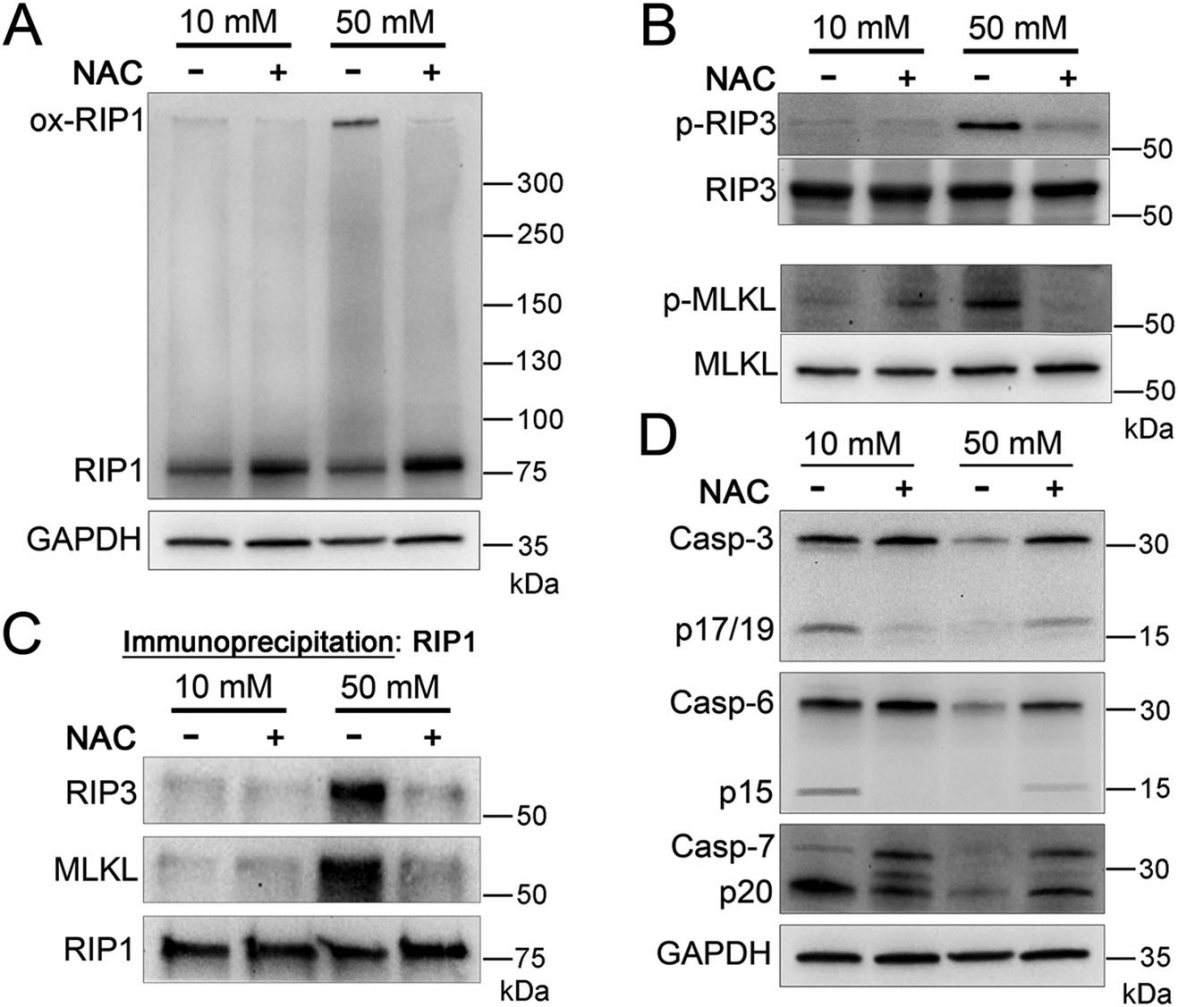
Activation of necrosome components and deactivation/loss of executioner caspases is driven by ROS in high-glucose conditions. **A** U937 cells were grown in 10 or 50 mM glucose followed by the treatment with TNF-α/CHX in the presence or absence of antioxidant, N-acetylcysteine (NAC), for 2.5 h followed by nonreducing SDS–PAGE and immunoblotting. A high MW, oxidized form of RIP1 is exhibited in 50 mM glucose, but is prevented by NAC. **B** U937 cells were treated as in **A** followed by reducing SDS–PAGE and immunoblotting. Total levels of RIP3 and MLKL were normalized. RIP3 and MLKL phosphorylation (p-RIP3 and p-MLKL, respectively) occurs in 50 mM glucose and is prevented by NAC. **C** U937 cells were treated as in **A** followed by immunoprecipitation of RIP1, reducing SDS–PAGE, and immunoblotting. RIP3 and MLKL co-precipitate with RIP1 in 50 mM glucose, but this is prevented by NAC. **D** U937 cells were treated as in **B**. Caspases-3, -6, and, -7 exhibit a decrease in activation and abundance in 50 mM that is prevented by NAC. All western blot images are representative of three independent experiments.

RIP1 is known to be oxidized by ROS, causing it to form a high MW oligomer necessary for its autophosphorylation^20^. To investigate if this occurs during the hyperglycemic shift to necroptosis, U937 monocytes were cultured in 10 or 50 mM glucose at 37 °C for 24 h, followed by the treatment with TNF-α/CHX in the presence or absence of NAC. Analysis of lysates separated by nonreducing SDS–PAGE and detected by western blot revealed the presence of oxidized RIP1 in 50 mM glucose conditions (Fig. 3A). This high MW RIP1 oligomer was inhibited by NAC in 50 mM glucose (Fig. 3A). Using conventional SDS–PAGE and western blot, we determined that total and phosphorylated levels of RIP3 and MLKL increased in 50 mM glucose conditions (Fig. 3B and Fig. S1A). The increase in total levels and phosphorylation, however, was prevented by the inhibition of ROS with NAC (Fig. 3B and Fig. S1A). Immunoprecipitation of RIP1 revealed that co-precipitation of RIP3 and MLKL in 50 mM glucose was prevented by NAC (Fig. 3C). Conversely, levels of executioner caspases-3, -6, and -7 decreased in 50 mM glucose, but rebounded upon ROS scavenging with NAC (Fig. 3D). These results suggest a key role for ROS in silencing apoptosis, while activating necroptosis in high-glucose conditions.

**Fig. 4:**
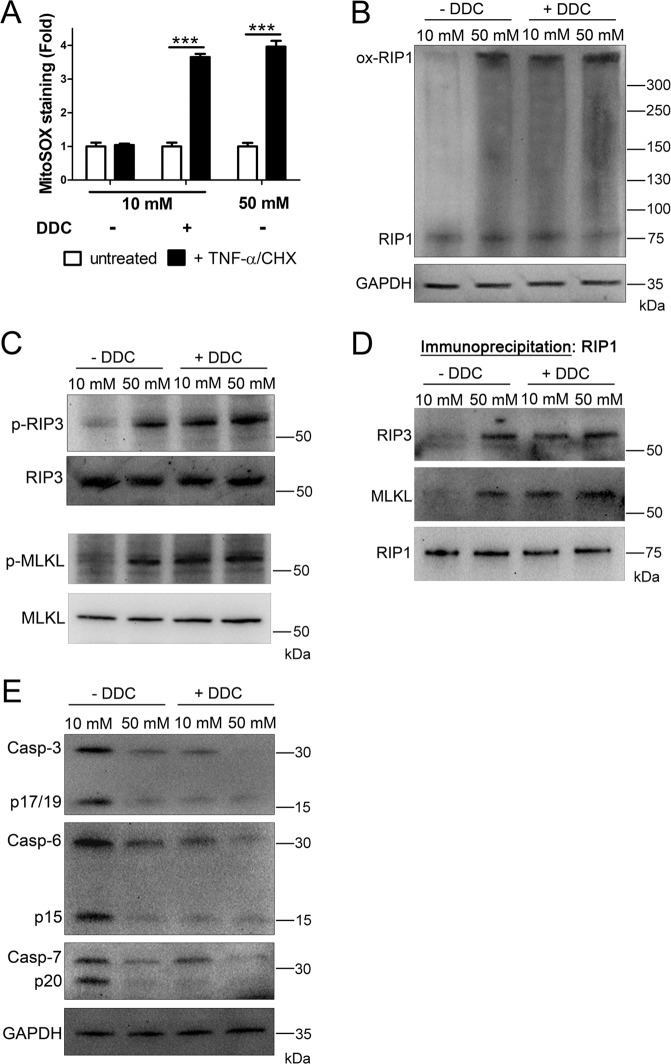
Glucose-independent induction of ROS causes the activation of necrosome components and deactivation/loss of executioner caspases. **A** U937 cells were grown in 10 or 50 mM glucose followed by the treatment with TNF-α/CHX in the presence or absence of superoxide dismutase inhibitor, diethyldithiocarbamate (DDC), for 4 h. Cells were then stained with MitoSOX Red and analyzed by flow cytometry. Glucose-independent induction of ROS with DDC produces a fold increase in MitoSOX staining similar to that exhibited in 50 mM glucose. Results are from three independent experiments. Graphed values represent mean ± standard deviation. Two-way ANOVA, ***p < 0.001. **B** U937 cells were grown in 10 or 50 mM glucose followed by the treatment with TNF-α/CHX in the presence or absence of DDC for 2.5 h followed by nonreducing SDS–PAGE and immunoblotting. RIP1 exists as a high MW, oxidized species in 50 mM glucose as well as in 10 or 50 mM glucose + DDC. **C** U937 cells were treated as in **B** followed by reducing SDS–PAGE and immunoblotting. Total levels of RIP3 and MLKL were normalized. RIP3 and MLKL are phosphorylated (p-RIP3 and p-MLKL, respectively) in 50 mM glucose and 10 or 50 mM glucose + DDC. **D** U937 cells were treated as in **B** followed by immunoprecipitation of RIP1, reducing SDS–PAGE, and immunoblotting. Both RIP3 and MLKL co-precipitate with RIP1 in 50 mM glucose and 10 or 50 mM glucose + DDC. **E** U937 cells were treated as in **C**. Caspases-3, -6, and -7 decrease in activation and abundance in 50 mM glucose and 10 or 50 mM glucose + DDC. All western blot images are representative of three independent experiments.

To probe the role of ROS further, we utilized the superoxide dismutase inhibitor, DDC, to induce ROS independent of cellular glucose levels (Fig. 4A). For this, U937 cells were cultured in 10 or 50 mM glucose for 24 h at 37 °C, followed by the treatment with TNF-α/CHX in the presence or absence of DDC. Analysis of lysates via nonreducing SDS–PAGE and western blot showed RIP1 oxidation in DDC-treated samples, regardless of the presence of 10 or 50 mM glucose (Fig. 4B). In addition, total levels and phosphorylation of RIP3 and MLKL increased following DDC treatment in 10 or 50 mM glucose (Fig. 4C and Fig. S1B). Immunoprecipitation of RIP1 showed that co-precipitation of RIP3 and MLKL occurred following DDC treatment in 10 or 50 mM glucose (Fig. 4D). Moreover, total and activated levels of executioner caspases-3, -6, and -7 decreased following treatment with DDC (Fig. 4E). Taken together, these results suggest and activity.

### Reactive oxygen species are necessary and sufficient to produce the shift from apoptosis to necroptosis

**Fig. 5:**
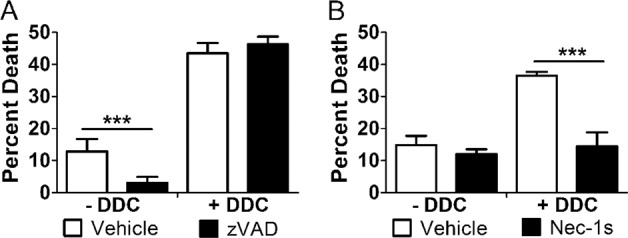
Glucose-independent induction of ROS produces a shift from apoptosis to necroptosis. U937 cells were grown in 10 mM glucose and treated with TNF-α/CHX in the presence or absence of superoxide dismutase inhibitor, diethyldithiocarbamate (DDC), for 24 h followed by WST-1 viability assay. **A** Cell death in the absence of DDC is prevented by the pan-caspase inhibitor, zVAD-fmk. Cell death in the presence of DDC is unaffected by zVAD-fmk. **B** Cell death in the presence of DDC is prevented by RIP1 inhibitor, necrostatin-1s (nec-1s). All results are from three independent experiments. Graphed values represent mean ± standard deviation. Two-way ANOVA, ***p < 0.001.

Our results thus far suggest a central role for ROS in promoting the hyperglycemic shift from apoptosis to necroptosis. To determine if ROS can promote this cell death shift independent of increased cellular glucose, U937 cells were cultured in 10 mM glucose for 24 h at 37 °C, followed by the treatment with TNF-α/CHX in the presence or absence of DDC. Cell viability was then measured and the impact of the RIP1 inhibitor, necrostatin-1s (nec-1s), and pan-caspase inhibitor, zVAD-fmk, was assessed. Treatment of U937 cells with TNF-α/CHX in the absence of DDC resulted in cell death that was prevented by zVAD-fmk (Fig. 5A). This is expected as TNF-α/CHX is a stimulus of apoptosis in normal glucose conditions^25^. Upon treatment with DDC, total cell death increased and could no longer be inhibited by zVAD-fmk (Fig. 5A). Conversely, the increased cell death seen in DDC conditions was prevented by nec-1s (Fig. 5B). These results indicate that cellular ROS are necessary and sufficient for the shift from apoptosis to necroptosis.

### Hyperglycemia primes the shift to necroptosis via ROS-induced activation of RIP1 and caspase loss prior to death receptor engagement

**Fig. 6:**
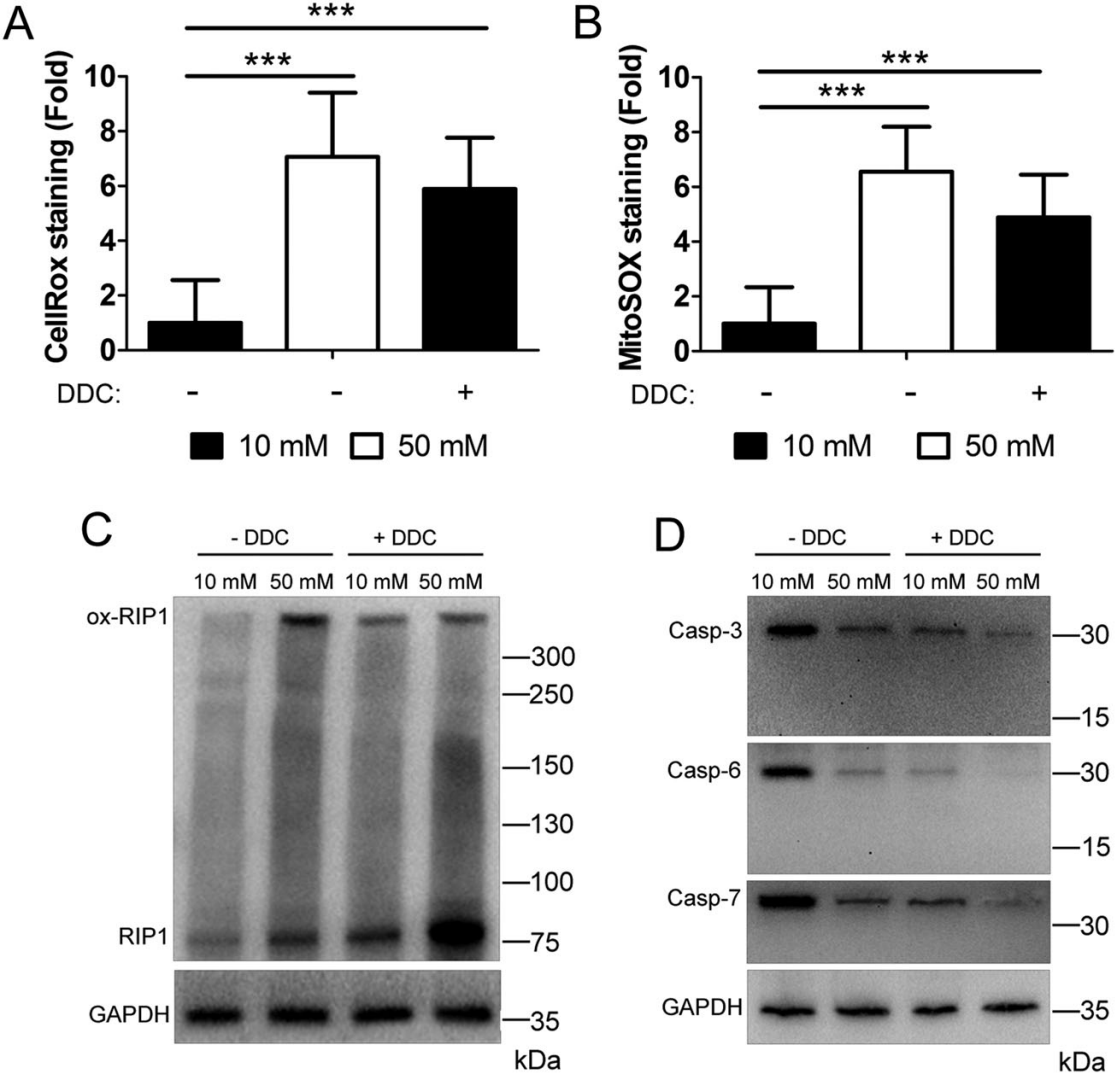
High glucose and ROS primes cells to undergo necroptosis, while preventing apoptosis in the absence of death receptor ligands. U937 cells were grown in 10 or 50 mM glucose in the presence or absence of superoxide dismutase inhibitor, diethyldithiocarbamate (DDC), for 24 h. Cells were then stained with **A** Cellrox Green or **B** MitoSOX Red, and analyzed by flow cytometry. There is a robust increase in staining for both reagents in cells grown in 50 mM glucose or those treated with DDC in normal glucose. Results in **A** and **B** are from three independent experiments. Graphed values represent mean ± standard deviation. Two-way ANOVA, ***p < 0.001. **C** U937 cells were grown in the presence or absence of DDC for 24 h followed by immunoblotting. RIP1 exists as a high MW, oxidized species in cells grown in 50 mM glucose or those treated with DDC. **D** U937 cells were treated as in **C**. Caspases-3, -6, and -7 decrease in cells grown in 50 mM glucose or treated with DDC. These results indicate that high glucose or ROS predispose cells to undergo necroptosis in the absence of TNF-α or other death receptor ligands. All western blot images are representative of three independent experiments.

Having established a role for ROS in promoting the hyperglycemic shift to necroptosis, we next determined the effect of high glucose on ROS production in the absence of death receptor stimulation by TNF-α/CHX. U937 cells were cultured in 10 or 50 mM glucose for 24 h, followed by the treatment with DDC for 2.5 h, staining with CellRox or MitoSOX reagents and flow cytometry. Even in the absence of TNF-α/CHX, there were robust increases in oxidative stress and mitochondrial ROS in 50 mM glucose or following treatment with DDC (Fig. 6A, B). As we observed increases in ROS in hyperglycemic conditions in the absence of TNF-α/CHX, we asked about the oxidation state of RIP1 in this situation. Indeed, RIP1 was oxidized to a high MW species in 50 mM glucose or following DDC treatment in the absence of stimulation by TNF-α/CHX (Fig. 6C). Moreover, levels of caspases-3, -6, and -7 decreased in 50 mM glucose or following DDC treatment in the absence of stimulation by TNF-α/CHX (Fig. 6D). Collectively, these results suggest that hyperglycemia primes cells for the shift from apoptosis to necroptosis via ROS-induced activation of RIP1 and loss of caspases.

### The hyperglycemic shift from apoptosis to necroptosis exacerbates neonatal HI-brain injury via mitochondrial ROS

**Fig. 7:**
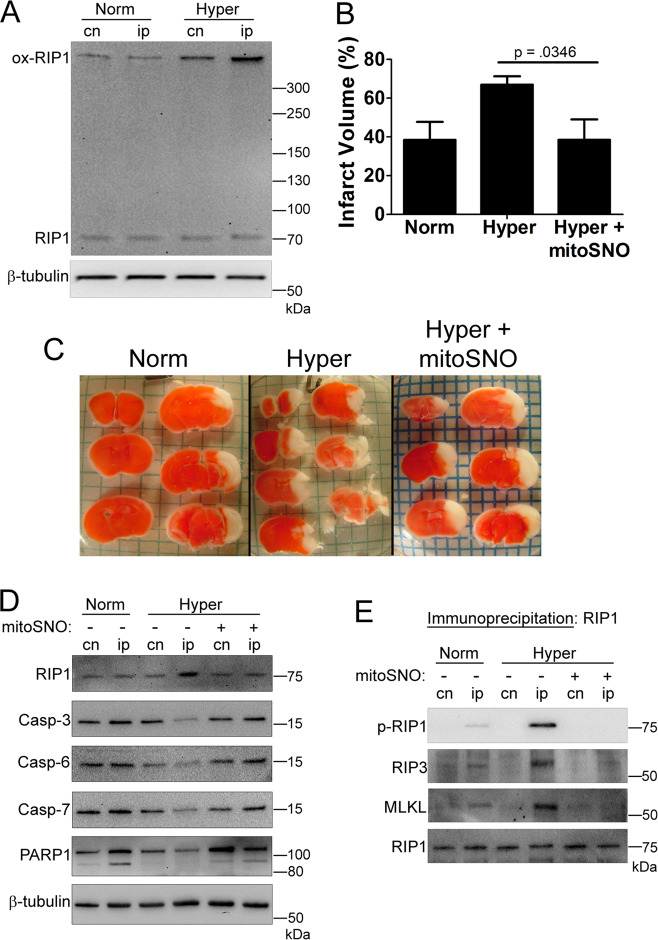
The hyperglycemic shift to necroptosis is driven by mitochondrial ROS during neonatal hypoxia-ischemia brain injury. Normal or hyperglycemic neonatal (p10) C57BL/6 mice were subjected to regional cerebral hypoxia-ischemia and sacrificed at 24 h of reperfusion. **A** Nonreducing SDS–PAGE and immunoblot of contralateral (cn) and ipsilateral (ip) tissue. There is a robust increase in the high MW, oxidized species of RIP1 in ipsilateral tissue from hyperglycemic mice. **B** Isolated brain tissue was stained with triphenly-tetrazolium (TTC) to measure infarct volume. Infarct volume is increased in hyperglycemic mice but is prevented by mitochondrial complex I deactivator, mitoSNO. *N* = 7 per mouse group. Graphed values represent mean ± standard deviation. One-way ANOVA with Fisher’s post hoc. **C** Representative images corresponding to quantitative results shown in **B**. **D** Reducing SDS–PAGE of contralateral and ipsilateral tissue. RIP1 increases while apoptotic markers, caspases-3, -6, -7, and PARP1 cleavage decrease in hyperglycemic mice. These trends are reversed by mitoSNO. **E** Immunoprecipitation of RIP1 from brain tissue. RIP1 is phosphorylated (p-RIP1) and RIP3 and MLKL co-precipitate with RIP1 in hyperglycemic mice. This did not occur in hyperglycemic mice treated with mitoSNO.

**Fig. 8:**
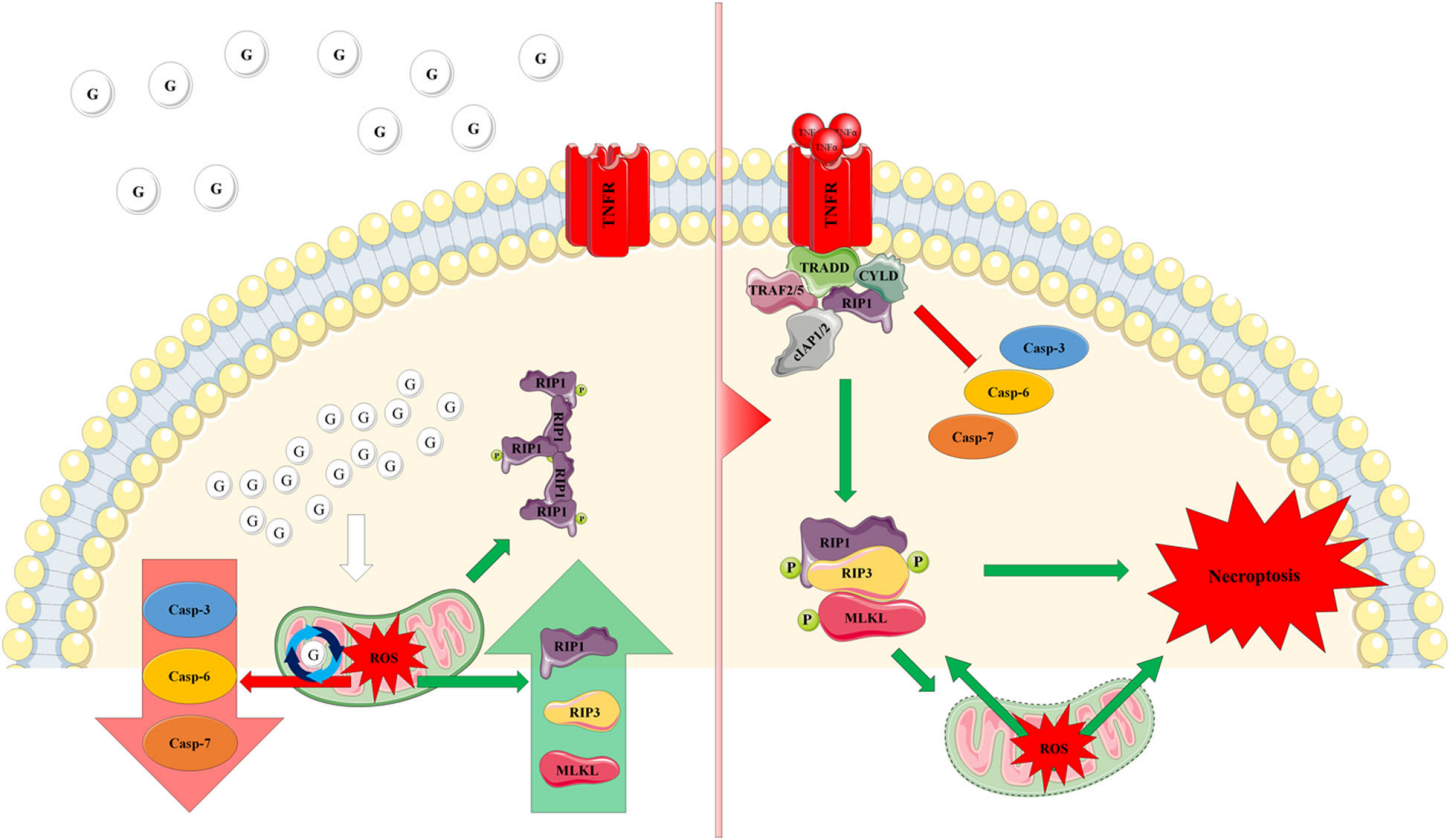
Model mechanism of the ROS-induced shift from apoptosis to necroptosis. In a hyperglycemic environment, high levels of cellular glucose lead to the production of mitochondrial ROS. Increased cellular ROS results in a decrease in the levels of executioner caspases with a concurrent increase in RIP1, RIP3, and MLKL, while also oxidizing RIP1 to a high MW oligomer. Once TNF-α engages TNFR, ROS promote formation of the necrosome and activation of RIP1, RIP3, and MLKL, while inhibiting activation of executioner caspases. In effect, the increased ROS silence apoptosis, while promoting necroptosis.

We have previously shown that the hyperglycemic shift from apoptosis to necroptosis occurs in hyperglycemic mice during neonatal HI-brain injury^5,29^. To determine the role of ROS in this cell death shift in vivo, we first detected oxidized RIP1 in brain homogenates. This high MW species of RIP1 increased in hyperglycemic mice undergoing neonatal HI-brain injury (Fig. 7A). Next, we determined the impact of mitochondria-targeted S-nitrosothiol (mitoSNO) on neonatal HI-brain injury in hyperglycemic mice. MitoSNO is a deactivator of mitochondrial complex I and thus ROS production^35^. Previously, it was shown that mitoSNO decreased ROS and prevented oxidative damage during neonatal HI-brain injury in normal mice^36^. Here, we show that administration of mitoSNO prevents exacerbation of infarct size seen in hyperglycemic mice undergoing neonatal HI-brain injury (Fig. 7B, C). Lysates from brain homogenates were analyzed via western blot, revealing an increase in total RIP1 in hyperglycemic mice that was prevented by mitoSNO (Fig. 7D). As seen previously, executioner caspases-3, -6, and -7 decreased in hyperglycemic mice undergoing neonatal HI-brain injury^29^. Administration of mitoSNO prevented this loss in caspases (Fig. 7D). In addition, PARP1 cleavage, a marker of apoptosis^25^, decreased in hyperglycemic mice, but rebounded upon mitoSNO treatment (Fig. 7D). When RIP1 was immunoprecipitated from hyperglycemic brain homogenates, RIP3 and MLKL co-precipitated, indicating necrosome formation (Fig. 7E). This co-precipitation was prevented following mitoSNO treatment (Fig. 7E). In agreement with this, RIP1 phosphorylation increased in hyperglycemic mice and was prevented by mitoSNO (Fig. 7E). Collectively, these results indicate that the hyperglycemic shift from apoptosis to necroptosis, which exacerbates neonatal HI-brain injury, depends on ROS (Fig. 8).

## Discussion

### ROS as regulators of cell death signaling

This work identifies ROS as important regulators of necroptosis, consistent with previous reports^14,20^. However, in this study, we have identified ROS as factors that regulate a switch from apoptosis to necroptosis. This occurs in a particular scenario: a hyperglycemic environment. TNF-induced apoptosis and necroptosis overlap in the first several steps of their signaling followed by a divergence of both pathways^1,8,9,25^. What regulates that divergence has been a topic of ongoing research. In the TNF-α pathways of apoptosis and necroptosis, this point of divergence is the formation of cytoplasmic complex II. It is at this step that either caspase-8 or RIP1 become activated depending upon whether apoptosis or necroptosis is occurring, respectively^1,8,9,25^. The formation of increased ROS as a result of exposure to high glucose is a key factor, which causes signaling to proceed in favor of necroptosis at this point of divergence. This hyperglycemic shift from apoptosis to necroptosis requires RIP1 in addition to ROS as this shift does not occur in its absence (Fig. 2B, C)^29^. While we have previously reported that high glucose leads to a shift from apoptosis to necroptosis^5,29^, the underlying mechanism remained unclear. Thus, this work reveals the roles of RIP1 and ROS in the mechanism of the hyperglycemic shift to necroptosis.

### Mechanism of the hyperglycemic shift from apoptosis to necroptosis

Identifying ROS and RIP1 as key factors in the hyperglycemic shift from apoptosis to necroptosis has allowed us to determine a model mechanism. In the presence of normal glucose, engagement of the TNF receptor (TNFR) by TNF-α leads to the activation of caspase-8 and downstream executioner caspases causing apoptosis^25^. In the presence of high glucose, mitochondrial ROS produce a shift from apoptosis to necroptosis by mediating the following molecular changes: (1) oxidization of RIP1 leading to formation of a high MW oligomer, (2) formation of the necrosome leading to phosphorylation of RIP1, RIP3, and MLKL, and (3) decrease in the levels and activation of executioner caspases-3, -6, and -7. As a result of these molecular changes, apoptosis is silenced and necroptosis ensues in its place. In addition, our work has revealed that oxidation of RIP1 and loss of executioner caspases occurs in response to high glucose in the absence of death receptor engagement (Fig. 6). This suggests that ROS prime hyperglycemic cells to undergo necroptosis once they are bound by TNF-α.

Previous publications have shown that oxidized RIP1 is sometimes associated with a shadowing between monomeric RIP1 and oxidized RIP1 (>300 kDa) on western blots^20^. Despite this shadowing, those publications identified a >300 kDa band at the top of the gel as oxidized RIP1 (ref. ^20^). Following their approach, we also identify a >300 kDa band as oxidized RIP1. We feel confident that the identity of this band is oxidized RIP1. We believe this as this high MW species was abolished by an antioxidant (Fig. 3A) and induced by ROS via inhibition of superoxide dismutases (Fig. 4B). The shadowing effect above monomeric RIP1 has also been observed on reducing western blots^37^, suggesting it may be artefactual.

### Translational relevance

This ROS-driven shift from apoptosis to necroptosis exacerbates neonatal HI-brain injury in the context of hyperglycemia (Fig. 7). In our experiments, we used mitoSNO^35^ which limits superoxide generation in the mitochondrial respiratory chain during reperfusion^36^. As mitoSNO protected against the hyperglycemia-driven exacerbation of neonatal HI-brain injury, this suggests a key role for mitochondrial ROS in this phenomenon. Moreover, our data suggests that ROS of mitochondrial origin mediate a shift from apoptosis to necroptosis during hyperglycemia (Fig. 7D, E). While the exact mechanisms for hyperglycemia-elevated ROS production in mitochondria is yet to be determined, it has been shown that during canonical necroptosis RIP1, RIP3, and MLKL translocate to the mitochondria and stimulate ROS production^38^. From an in vivo perspective, cerebral ischemia is associated with a dramatic increase in succinate accumulation^36,38^. Upon reperfusion, this accumulated succinate leads to excessive ROS release in mitochondrial complex I via reverse electron transfer^38^. MitoSNO transiently inhibits complex I reactivation during reperfusion and, therefore, significantly limits reverse electron transfer and the rate of superoxide production^35,36^. This suggests a possible role for reverse electron transfer in this ROS-driven shift from apoptosis to necroptosis during hyperglycemia.

## Materials and methods

### Cell culture

U937 monocytes were obtained from ATCC (Manassas, VA, USA) and were cultured in RPMI 1640 medium supplemented with 10% fetal bovine serum at 37 °C and 5% CO_2_. *rip1* CRISPR KO and NTC U937 cell lines were generated using the CRISPR-Cas9 lentiviral system, as previously described^29^. NTC and *rip1* CRISPR KO cells were cultured with 3 µg/mL puromycin for selection under the same conditions as the U937 wild-type cells. All cell lines have been recently authenticated and are free of *Mycoplasma* contamination.

### Inhibitors and ROS scavengers

Pharmacological inhibitors were incubated with the cells at 37 °C and 5% CO_2_ for 1 h prior to the addition of TNF-α at the following concentrations: VAS-2870 (5 μM), CCCP (10 μM), DDC (1 μM), nec-1s (50 μM), and zVAD-fmk (50 μM). ROS scavengers were similarly incubated prior to the addition of TNF-α at the following concentrations: catalase (100 units/mL), NAC (10 mM) and mitoTEMPO (500 μM). All inhibitors and ROS scavengers were purchased from Millipore-Sigma.

### Preparation of cell lysates

U937 wildtype, NTC, and *rip1* CRISPR KO cells were cultured in media supplemented with 10 or 50 mM glucose for 24 h at 37 °C and 5% CO_2_. Following this, they were treated with 1 ng/mL TNF-α and 0.25 µg/mL cycloheximide for 2.5 h. Cells were washed with PBS and lysed with Tris-buffered saline (TBS) supplemented with 1% triton X-100, 1 mM phenylmethylsulfonyl fluoride, 1 mM sodium orthovanadate, and 1× ProBlock™ Gold (Goldbio) for 30 min on ice. Lysates were clarified by centrifugation at 20,000 × *g* for 20 min at 4 °C. For some experiments, cells were cultured in 10 or 50 mM glucose for 24 h followed by lysate preparation. For other experiments, cells were cultured in 10 or 50 mM glucose for 24 h followed by the treatment with DDC for 2.5 h and lysate preparation. All reagents were obtained from Millipore-Sigma unless indicated otherwise.

### Immunoprecipitations

Immunoprecipitation of RIP1 or RIP3 was performed on U937, *rip1* CRISPR KO, or NTC cell lysates prepared, as detailed above. Following lysate creation, supernatants were precleared with agarose beads, and incubated with 10 µg of anti-human RIP1 (BD Biosciences) or RIP3 (Cell Signaling Technology) added to supernatants and allowed to incubate with gentle mixing overnight at 4 °C. Supernatants were then incubated with Protein G Plus agarose beads (Pierce) for 2 h at room temperature. Beads were washed thoroughly, resuspended in 1× Laemmli buffer, run on SDS–PAGE, and western blotted.

### Cell death assays

Cells were incubated for 24 h in RPMI 1640 media containing 10 or 50 mM glucose at 37 °C and 5% CO_2_. Cells were adjusted to 1 × 10^6^ cells/mL in fresh media and treated with 0.4 lytic units of TNF-α and 0.25 µg/mL cycloheximide for 24 h. The lytic units of TNF-α have been described previously^29^. Pharmacological inhibitors and ROS scavengers were added where indicated at the concentrations listed above. For each WST-1 assay *n* = 6. This *n* was chosen based on previous experiments^5,29^. WST-1 assays were performed in three independent experiments. WST-1 reagent was used to measure cell death according to the manufacturer’s instructions (Takara). Using WST-1, percent viability was calculated as follows:

$${\mathrm{\% Viability = 100 \times }}\frac{{{\mathrm{absorbance}}\;{\mathrm{of}}\;{\mathrm{TNF\alpha}} {\mathrm{/CHX}}\;{\mathrm{treated}}\;{\mathrm{cells}}}}{{{\mathrm{absorbance}}\;{\mathrm{of}}\;{\mathrm{negative}}\;{\mathrm{control}}\;{\mathrm{cells}}}}.$$

### Flow cytometry (CellROX and MitoSOX)

For flow cytometry analyses, 10,000 events were collected for each sample after gating out debris. Sample data were collected utilizing a BD FACSVerse flow cytometer. Data files were analyzed using FlowJo V10. Prior to analysis, cells (U937, *rip1* CRISPR KO, and NTC) were incubated in 10 or 50 mM glucose for 24 h at 37 °C and 5% CO_2_. Cells were treated with 1 ng/mL TNF-α at 37°C and 5% CO_2_ for 4 h. For ROS detection, CellROX Green reagent or MitoSOX Red superoxide indicator (Invitrogen) were added to cells at a final concentration of 5 μM and used according to the manufacturer’s instructions. For some experiments, cells were cultured in 10 or 50 mM glucose for 24 h followed by staining and flow cytometry. For other experiments, cells were cultured in 10 or 50 mM glucose for 24 h followed by the treatment with DDC for 4 h, and staining and flow cytometry. For each flow cytometry experiment *n* = 6. This *n* was chosen based on previous experiments^29^. Flow cytometry experiments were performed in three independent experiments.

### Western blots

Lysates, immunoprecipitates, and tissue homogenates were separated via SDS–PAGE, with and without the inclusion of reducer (2-mercaptoethanol), transferred to a PVDF membrane, and blocked in TBS-T buffer containing either 5% milk or BSA (for phosphorylated proteins) for 30 min at room temperature. The blots were incubated with primary antibody diluted in block buffer overnight at 4 °C. All primary antibodies were obtained from Cell Signaling Technology, unless otherwise indicated. Primary antibodies were used at the following dilutions: anti-human GAPDH (1:10000), anti-human caspase-3 (1:1000), anti-human/mouse caspase-6 (1:1000), anti-human/mouse caspase-7 (1:1000), anti-human RIP1 (1:1000), anti-human p-RIP1 (1:1000), anti-human RIP3 (LSBio, 1:1000), anti-human p-RIP3 (1:1000), anti-human MLKL (1:1000), anti-human p-MLKL (1:1000), anti-mouse β-tubulin (1:1000), anti-mouse caspase-3 (1:1000), anti-mouse caspase-6 (1:1000), anti-mouse caspase-7 (1:1000), anti-mouse PARP1 (1:1000),anti-mouse RIP1 (1:1000), anti-mouse p-RIP1 (1:1000), anti-mouse RIP3 (1:1000), and anti-mouse MLKL (1:1000). Following overnight incubation, blots were washed with TBS-T containing 5% milk (or BSA), incubated with secondary HRP conjugate antibodies for 1 h at room temperature. After the final wash, blots were developed with chemiluminescent substrate and read in a Bio-Rad ChemiDoc XRS+. All western blots are the results of three independent experiments.

### In vivo brain hypoxia-ischemia model

All studies were conducted according to a protocol approved by the Columbia University Institutional Animal Care and Use Committee and in accordance with the Association for Assessment and Accreditation of Laboratory Animal Care guidelines. Hyperglycemia was induced in male and female neonatal (p10) C57BL/6 J mice subjected to regional HI-brain injury according to our description in ref. ^5^. Immediately after HI, mice were administered with either MitoSNO (500 ng/kg) or vehicle (DMSO) intranasally, 1 μl aliquots (10 μl total), over 40 min. At 24 h of reperfusion, all mice were sacrificed and the extent of brain injury was estimated using triphenly-tetrazolium (TTC) staining. Infarct volume was expressed as % of the hemisphere ipsilateral to the carotid artery ligation side. Brain tissue was also used to create homogenates which were analyzed by immunoprecipitation, SDS–PAGE, and western blot. *N* = 7 per mouse group. This *n* was chosen based on power analysis and previous experiments^5^. For TTC staining and analysis, investigators were blinded (unaware of sample identity), effectively randomizing the samples.

### Statistical analysis

Two-way ANOVA with Bonferroni posttest was used to analyze significance of all quantitative in vitro data. One-way ANOVA test with Fisher’s post hoc analysis was used to determine difference in cerebral infarct volume in vivo. All results are from three or more independent experiments. Statistics were calculated using GraphPad Prism 5.0.

## Supplementary information

Figure S1

Supplemental material
