# Supplementary figures and images for: Mitochondrial ROS prime the hyperglycemic shift from apoptosis to necroptosis
Source: Cell Death Discov. 2020 Nov 26;6:132. doi: 10.1038/s41420-020-00370-3 (PMC7693268; doi:10.1038/s41420-020-00370-3)

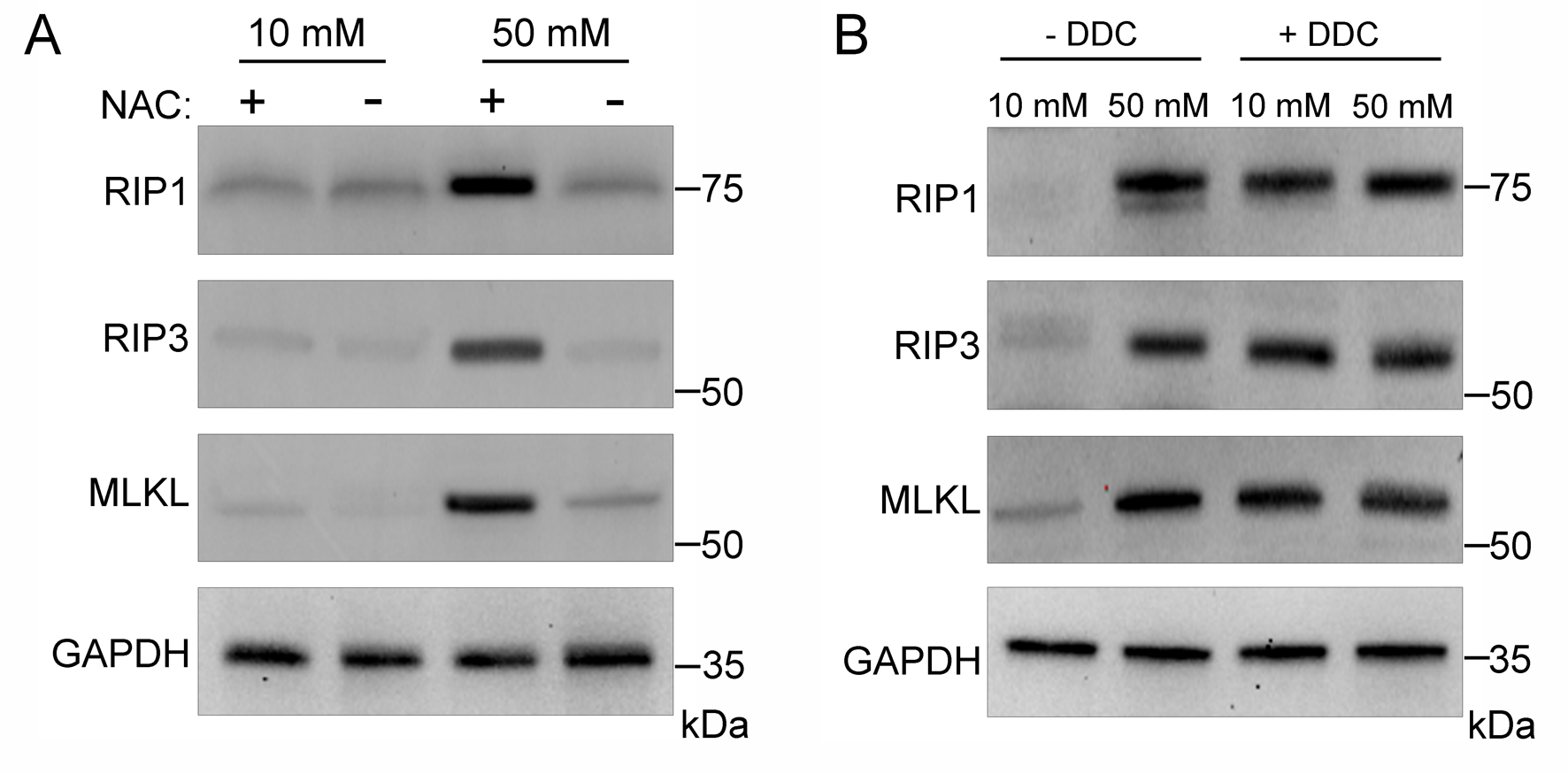

Supplement: Supplementary file 1 — Figure S1 [file 41420_2020_370_MOESM1_ESM.tif]
