# Supplementary material for: Mitochondrial ROS prime the hyperglycemic shift from apoptosis to necroptosis
Source: Cell Death Discov. 2020 Nov 26;6:132. doi: 10.1038/s41420-020-00370-3 (PMC7693268; doi:10.1038/s41420-020-00370-3)
Supplement: Supplementary file 2 — Supplemental material [file 41420_2020_370_MOESM2_ESM.docx]

**Figure S1: Total levels of RIP1, RIP3, and MLKL increase in response to ROS. A.)** U937 cells were grown in 10 or 50 mM glucose followed by treatment with TNF-α/CHX in the presence or absence of antioxidant, N-acetylcysteine (NAC), for 2.5 h followed by SDS-PAGE and immunoblotting. Total levels of RIP1, RIP3, and MLKL increase in 50 mM glucose but this is prevented by NAC. These blots correspond to **Figures 2D and 3B**. **B.)** U937 cells were grown in 10 or 50 mM glucose followed by treatment with TNF-α/CHX in the presence or absence of DDC for 2.5 h followed by SDS-PAGE and immunoblotting. Total levels of RIP1, RIP3, and MLKL increase in 50 mM glucose and 10 or 50 mM glucose + DDC. These blots correspond to **Figures 2E and 4C**. All western blot images are representative of 3 independent experiments.
